# Platinum‐ and CuO_*x*_‐Decorated TiO_2_ Photocatalyst for Oxidative Coupling of Methane to C_2_ Hydrocarbons in a Flow Reactor

**DOI:** 10.1002/anie.202007557

**Published:** 2020-07-16

**Authors:** Xiyi Li, Jijia Xie, Heng Rao, Chao Wang, Junwang Tang

**Affiliations:** ^1^ Solar Energy and Advanced Materials Research Group Department of Chemical Engineering University College London Torrington Place London WC1E 7JE UK; ^2^ State Key Laboratory of Inorganic Synthesis and Preparative Chemistry College of Chemistry Jilin University 2699 Qianjin Street Changchun 130012 China; ^3^ International Center of Future Science Jilin University 2699 Qianjin Street Changchun 130012 China

**Keywords:** C_2_ hydrocarbons, flow reactors, methane conversion, oxidative coupling of methane (OCM), photocatalysis

## Abstract

Oxidative coupling of methane (OCM) is considered one of the most promising catalytic technologies to upgrade methane. However, C_2_ products (C_2_H_6_/C_2_H_4_) from conventional methane conversion have not been produced commercially owing to competition from overoxidation and carbon accumulation at high temperatures. Herein, we report the codeposition of Pt nanoparticles and CuO_x_ clusters on TiO_2_ (PC‐50) and use of the resulting photocatalyst for OCM in a flow reactor operated at room temperature under atmospheric pressure for the first time. The optimized Cu_0.1_Pt_0.5_/PC‐50 sample showed a highest yield of C_2_ product of 6.8 μmol h^−1^ at a space velocity of 2400 h^−1^, more than twice the sum of the activity of Pt/PC‐50 (1.07 μmol h^−1^) and Cu/PC‐50 (1.9 μmol h^−1^), it might also be the highest among photocatalytic methane conversions reported so far under atmospheric pressure. A high C_2_ selectivity of 60 % is also comparable to that attainable by conventional high‐temperature (>943 K) thermal catalysis. It is proposed that Pt functions as an electron acceptor to facilitate charge separation, while holes could transfer to CuO_x_ to avoid deep dehydrogenation and the overoxidation of C_2_ products.

## Graphical abstract

**Teamwork**: TiO_2_ decorated with both platinum nanoparticles and CuO_*x*_ clusters enabled photocatalytic oxidative coupling of methane in a flow system at room temperature and atmospheric pressure with a high yield rate of 6.8 μmol h^−1^ for the selective synthesis of C_2_ hydrocarbons. It is proposed that Pt functions as an electron acceptor to facilitate charge separation, while holes may transfer to CuO_x_ to avoid deep dehydrogenation and overoxidation (see picture).

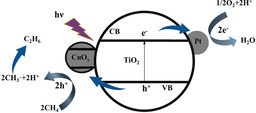

Under the pressure of the decreasing reserves of crude oil, natural gas (methane) is widely accepted as an alternative for fuel and more importantly as a fundamental building block for chemical synthesis.^1^ So far, only indirect conversion of methane via syngas (a certain ratio of H_2_ and CO) process reaches a feasible commercial scale.^2^ This multistage process is not only energy‐intensive, operating at a high temperature with a high capital cost, but also accompanied by substantial CO_2_ emission. Therefore, there are manifest financial and environmental incentives to explore the direct transformation of methane into value‐added chemicals under moderate conditions.

Among various direct transformation technologies, oxidative coupling of methane (OCM) to give ethane and ethylene has been regarded as a promising route for the valorisation of methane.^3^ However, it is difficult to activate or convert CH_4_ owing to its inert nature, including its high C−H bond energy (439 kJ mol^−1^), symmetrical tetrahedral geometry, and low polarizability (2.84×10^−40^ C^2^ m^2^ J^−1^).^4^ The introduction of oxygen and a high temperature are thus conventionally required to overcome the thermodynamic barriers and increase the conversion. Such reaction conditions inevitably produce the undesired while thermodynamically favourable products, CO_2_ and graphitic carbon. The resulting low selectivity and low yield of C_2_ products brings about a barrier to commercialization.

Photocatalysis, employing photons under mild conditions instead of thermal energy, has been regarded as a potential economic technology to break the thermodynamic barrier to the direct conversion of methane. Thus, harsh reaction conditions, overoxidation, and the deposition of coke could be theoretically avoided. In the past two decades, a wide range of products have been successfully obtained through photocatalytic methane conversion, such as methanol,^5^ ethanol,^6^ ethane/ethylene,^7^ benzene,^8^ and syngas,^9^ in batch reactors, but with very moderate efficiency due to the following major causes. First, the high recombination rate of photoinduced carriers in the intrinsic semiconductor greatly limits their quantum efficiency, thus resulting in low conversion. Next, the pristine photocatalysts with an unmodified interface lead to poor selectivity because of overoxidation by the extremely oxidative photoholes in the valence band (VB) of the photocatalyst and the lack of active centres. More importantly, the majority of photocatalytic methane conversion reactions were carried out in batch reactors. Such reactions are easy to carry out, but it is theoretically hard to avoid overoxidation as the long residence time in the batch reactor favours the thermodynamically stable product CO_2_. In addition, such a system is also challenging for scale‐up.

In this study, the comodification of TiO_2_ (PC‐50) photocatalysts by Pt nanoparticles and CuO_*x*_ clusters was investigated to overcome the major drawbacks mentioned above for photocatalytic OCM. Furthermore, a flow system was applied to manipulate the residence time of the reactants at room temperature and atmospheric pressure. The synergy of Pt and Cu species on PC‐50 led to an increased C_2_ (ethane and ethylene) yield (6.8 μmol h^−1^), which was approximately 3.5 times higher than that observed with the parent semiconductor PC‐50. It is also the highest yield for C_2_ products among all the photocatalytic methane conversion processes reported under atmospheric pressure. The C_2_ selectivity of 60 % was comparable to that for traditional thermal catalysis at high temperature (>943 K). The active species were then investigated by X‐ray photoelectron spectroscopy (XPS), transmission electron microscopy (TEM), electronic paramagnetic resonance (EPR), photoluminescence (PL) spectroscopy, transient photocurrent response, and in situ EPR.

TiO_2_ has been regarded as one of the benchmark photocatalysts owing to its intrinsically high stability and activity under UV photons. Thus, commercial anatase TiO_2_ (PC‐50) was selected as a starting substrate. Then, Pt nanoparticles and CuO_x_ species were introduced by photodeposition and subsequent wet impregnation (see the Supporting Information for details). The as‐prepared sample was designated as Cu_*x*_Pt_*y*_/PC‐50, in which *x* and *y* represent the nominal weight ratio of Cu and Pt to PC‐50, respectively. Cu_0.1_/PC‐50, Pt_0.5_/PC‐50, and PC‐50 were the reference samples.

The crystal structures of all the as‐prepared samples were indexed to anatase TiO_2_ (JCPDS no. 84‐1286), as shown in powder X‐ray diffraction (PXRD) spectra (Figure 1 a). After the introduction of Pt and Cu, the PXRD spectra remained unchanged, indicating a stable framework. Additionally, the spectra displayed no extra peaks for copper or platinum species, most likely because of their low amount and/or high dispersion.^10^

**Figure 1:**
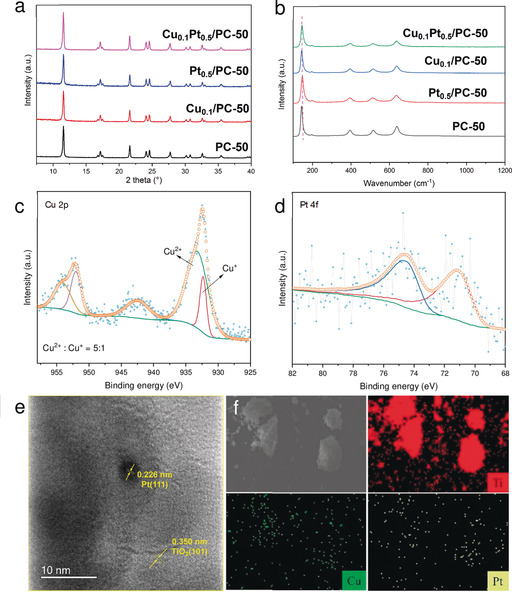
a) PXRD and b) Raman spectra of Cu_0.1_Pt_0.5_/PC‐50, Pt_0.5_/PC‐50, Cu_0.1_/PC‐50, and PC‐50. c) Cu 2p XPS spectra of Cu_2.0_/PC‐50. d) Pt 4f XPS spectra of Cu_0.1_Pt_0.5_/PC‐50. e) HR‐TEM image of Cu_0.1_Pt_0.5_/PC‐50. f) EDX elemental mapping (Ti, Cu, and Pt) of Cu_0.1_Pt_0.5_/PC‐50.

The anatase structure was further supported by Raman spectroscopy (Figure 1 b). The typical Raman peaks for anatase TiO_2_ were clearly observed at 144 (E_g_), 198 (E_g_), 399 (B_1g_), 512 (A_1g_), and 639 cm^−1^ (E_g_).^11^ Notably, a slight blue shift and broadening of the 144 cm^−1^ Raman peak was observed after the introduction of cocatalysts, in particular Cu_0.1_Pt_0.5_/PC‐50. This change in the peak could be attributed to surface strain after surface modifications.^12^

The photoabsorption properties of the as‐prepared samples were investigated by ultraviolet–visible diffuse reflectance spectroscopy (UV/Vis DRS). After the introduction of CuO_*x*_ clusters, the photoabsorption was enhanced in the range from 200 to 320 nm (Figure 2 a), most likely because of charge transfer between oxygen and isolated copper(II) species and the charge transfer in clusters.^13^ The absorption edge remained almost unchanged for all of the samples, thus indicating the intact band structure of PC‐50 and the little contribution from CuO_*x*_ absorption.

**Figure 2:**
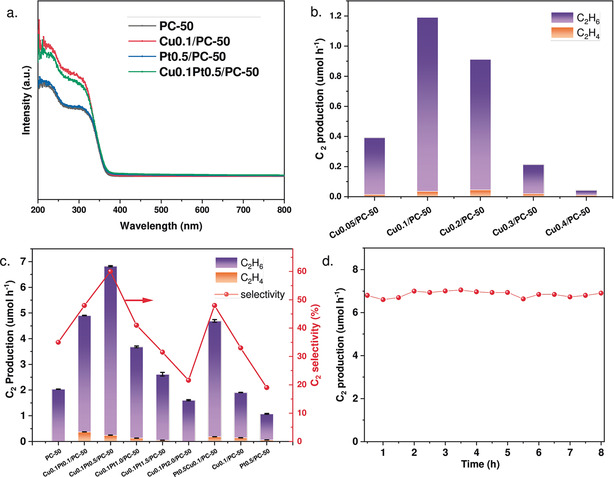
a) UV‐DRS spectra of Cu_0.1_Pt_0.5_/PC‐50, Pt_0.5_/PC‐50, Cu_0.1_/PC‐50, and PC‐50. b) C_2_ production of photocatalytic OCM over Cu_*x*_/PC‐50 (*x*=0.05, 0.1, 0.2, 0.3, 0.4; reaction conditions: O_2_/CH_4_=1:240, GHSV=1200 h^−1^, 10 % of CH_4_, 365 nm LED 20 W, 40 °C). c) C_2_ production and selectivity of photocatalytic OCM over Cu_0.1_Pt_y_/PC‐50 (*y*=0.1, 0.5, 1.0, 1.5, 2.0 wt %), Cu_0.1_/PC‐50, PC‐50, Pt_0.5_/PC‐50, and Pt_0.5_Cu_0.1_/PC‐50 (reaction conditions: O_2_/CH_4_=1:400, GHSV=2400 h^−1^, 10 % of CH_4_, 365 nm LED 40 W, 40 °C). d) Stability test of photocatalytic OCM over Cu_0.1_Pt_0.5_/PC‐50. GHSV=gas hourly space velocity.

The photocatalytic activity of the as‐prepared samples for OCM was evaluated in a flow system at room temperature and under atmospheric pressure. It has been widely reported that photoinduced holes at the valence band of TiO_2_ tended to promote the mineralization of CH_4_ into CO_2_ through deep dehydrogenation.^14^ The valence band edge of CuO and Cu_2_O is around 0.75 and 0.99 eV more negative (vs. NHE), respectively, as compared to TiO_2_.^15^ This more negative valence band edge indicated the potential formation of C_2_ products rather than CO_2_ after the introduction of copper species, because copper species were expected to accept the photoinduced holes from TiO_2_ and dramatically lower their oxidation potential. Moreover, Cu^II^ clusters as active sites have previously been observed to selectively oxidise methane in thermal catalysis.^16^, ^17^ The optimum content of copper was first investigated (Figure 2 b). It exhibited a volcanic trend with an increasing weight percentage of Cu and reached the highest C_2_ yield over Cu_0.1_/PC‐50 (1.2 μmol h^−1^). This trend was probably observed because an excessive amount of CuO could act as a recombination centre of photoinduced electrons and holes,^18^ as further discussed later.

After the Cu amount had been optimised, Pt was added to facilitate charge separation as a widely known electron acceptor.^19^ To test the photocatalytic efficiency under relatively harsh conditions, we increased the space velocity from 1200 to 2400 h^−1^ and then investigated bimetallic cocatalyst samples (Figure 2 c; see also Figure S8). The conversion of methane was increased as compared to the use of pristine TiO_2_, while the yield of both C_2_ products and CO_2_ increased after the codeposition of Pt nanoparticles and CuO_*x*_ clusters. This result was due to more available separated photoinduced carriers through the efficient transfer of electrons and holes to Pt and CuO_x_ clusters, respectively. The selectivity for C_2_ products first increased as compared to selectivity for CO_2_ as the amount of Pt on the Pt‐ and CuO_*x*_‐coloaded samples increased. However, over‐increasing the amount of Pt caused a reduction in both yield and selectivity for C_2_ products. The yield of C_2_ products on the optimised sample Cu_0.1_Pt_0.5_/PC‐50 was 6.8 μmol h^−1^, which is more than twice as high the sum of the yields on Pt_0.5_/PC‐50 (1.07 μmol h^−1^) and Cu_0.1_/PC‐50 (1.9 μmol h^−1^), thus indicating the importance of the synergistic effect. Moreover, the yield of CO_2_ only increased by around 20 % as compared to that on PC‐50, indicating the indispensable role of CuO_x_ clusters in shifting selectivity towards C_2_. Remarkably, this yield is about four times higher than the reported production rate of C_2_H_6_ and C_2_H_4_ by photocatalytic methane conversion with >300 nm irradiation over different catalysts under atmospheric pressure (see Table S2 in the Supporting Information). Given that some reactions in Table S2 were non‐oxidative coupling of methane, their yields were relatively low owing to the high thermodynamic barriers.^20^ The yield of C_2_ products over our optimised sample was also higher than for the partial oxidation of methane. Furthermore, the selectivity towards C_2_ products of 60 % was comparable to that of traditional catalysts (e.g. Li/MgO) operated at high temperature (>943 K).^3^, ^21^

We also calculated the apparent quantum yield (AQE) based on methane conversion for Cu_0.1_Pt_0.5_/PC‐50 and PC‐50. The AQE of Cu_0.1_Pt_0.5_/PC‐50 (0.5 % at 365 nm) was nearly twice as high as that of PC‐50 (0.25 % at 365 nm), indicating the higher utilization of light energy. The further addition of Pt led to decreased C_2_ selectivity and increased CO_2_ yield, with the highest CO_2_ yield reaching 11.6 μmol h^−1^. We believe too many Pt nanoparticles might lead to the excessive formation of O_2_^.−^, which was the major component for overoxidation.^22^ Accordingly, Pt_0.5_/PC‐50 only exhibited an increased yield of CO_2_, but the lowest yield of C_2_ products as compared to PC‐50. This activity resulted in the highest selectivity for CO_2_ (ca. 80 %), again due to the increased availability of photoinduced electrons for O_2_^.−^ generation and strong oxidative holes at the valence band of TiO_2_.

The preparation order of the two cocatalysts was changed to observe its effect. Another photocatalyst, Pt_0.5_Cu_0.1_/PC‐50, was thus prepared. Interestingly, it showed a decreased C_2_ yield (4.7 μmol h^−1^) as compared to Cu_0.1_Pt_0.5_/PC‐50, indicating that the deposition sequence of cocatalysts also had an important influence on their performance. The function of Pt was believed to be to accept photoinduced electrons and help charge separation. If the CuO_*x*_ clusters were deposited first, some of them would block the contact between Pt particles and TiO_2_, leading to a reduced charge‐separation effect, thus lowering the conversion and yield.

It was noted that the yield of C_2_ products over Cu_0.1_/PC‐50 was lower than that over PC‐50, while the yield of CO_2_ was similar. As proved by the XPS results later, the copper species in our samples was mainly CuO. Its conduction band (CB) was 0.75 eV more positive than that of TiO_2_. Taking into account the more negative VB of CuO than that of TiO_2_, some holes transferred from the VB of TiO_2_ would recombine with the electrons from the CB of TiO_2_ on the CuO_*x*_ clusters. This could lead to the decreased generation of methyl radicals, which would have a more negative effect on the coupling to C_2_ species than deep oxidation to CO_2_ because of the second‐order nature of the coupling reaction to C_2_ products.^23^ Some remaining highly oxidative holes with the O_2_^.−^ formed by the remaining electrons continued to proceed the overoxidation of methane to CO_2_. Thus, the yield of CO_2_ exhibited nearly no change and the yield of C_2_ products decreased after the single introduction of CuO_*x*_ clusters, indicating the important role of Pt nanoparticles for the synergistic effect.

In our system, only ethane, ethylene, and CO_2_ could be detected as products by our GC equipped with a methaniser unit and an FID detector (see Figure S7). Thus, the C_2_ selectivity mentioned above was calculated based on the measured products. No products could be detected when the reaction was carried out in the absence of methane or without light irradiation (see Table S1). These results confirmed that it was a photocatalytic process with CH_4_ as the only carbon source.

The stability of the optimised sample Cu_0.1_Pt_0.5_/PC‐50 was then tested. No decay of C_2_ yield except slight fluctuation could be observed during an 8 h reaction (Figure 2 d). The structure of catalysts and the chemical states of active species also remained unchanged during the reaction (see Figures S5 and S6). These results indicated that Cu_0.1_Pt_0.5_/PC‐50 exhibited excellent stability in the photocatalytic OCM process.

XPS was then conducted to analyse the chemical states of cocatalysts on the optimum catalyst (Figure 1 c,d; see also Figures S2 and S3). Due to the extremely low loading amount of copper species, no clear Cu 2p peak was observed on Cu_0.1_Pt_0.5_/PC‐50 (see Figure S3). Thus, a sample (Cu_2.0_/PC‐50), prepared by the same procedure but with a large loading amount of copper species was used to identify the chemical states of Cu on PC‐50 (Figure 1 c). The peaks attributed to Cu 2p_3/2_ and Cu 2p_1/2_ at around 933.4 and 953.9 eV, coupling with the shake‐up satellite peak at around 942.6 eV, indicated the main existence of fully oxidised CuO.^24^ In addition, a small amount of Cu^I^ (Cu^II^/Cu^I^=5:1) could be found with peaks at 932.2 and 952 eV, respectively. It is believed that similar species were formed on the best sample, Cu_0.1_Pt_0.5_/PC‐50. As compared with PC‐50 and Pt_0.5_/PC‐50, the binding energy of the Ti 2p_3/2_ transition shifted to lower binding energy over Cu_0.1_/PC‐50 and Cu_0.1_Pt_0.5_/PC‐50 (see Figure S2). The lower binding energy suggested the electrons probably transferred from Cu to Ti, thus indicating the interaction between the cocatalysts and PC‐50.^25^ XPS analysis of Pt provided peaks at 71.2 and 74.6 eV, which were assigned to metallic states.^26^

TEM and HRTEM images provided further information on the particle size and distribution of Cu_0.1_Pt_0.5_/PC‐50. Some nanoparticles were dispersed on PC‐50 with diameters from 3.5 to 6 nm (see Figure S4). These nanoparticles were further identified by HRTEM (Figure 1 e), in which the *d* spacing of lattice fringes could be attributed to Pt (111, 0.226 nm) and anatase TiO_2_ (101, 0.350 nm).^27^ The copper species were not observed at this resolution, suggesting the existence of smaller clusters. Energy‐dispersive X‐ray (EDX) mapping showed that Cu and Pt were dispersed homogeneously (Figure 1 f), in good agreement with the XRD results.

To further unravel the chemical state of copper species and the charge transfer, in situ EPR was carried out (Figure 3 a). As compared with Pt_0.5_/PC‐50, Cu_0.1_Pt_0.5_/PC‐50 exhibited new spectra corresponding to CuO hyperfine structure owing to *I*=3/2 of Cu^II^, indicating the existence of Cu^II^ in the copper species.^28^ Although the existence of long‐range dipolar interactions between different Cu^II^ sites resulted in the broadening of spectral lines, the anisotropic hyperfine structure could be found after careful analysis: *g*_∥_=2.395 with A_∥_≈100 G was obtained, whereas the value for *g*_⊥_=2.05 could not be resolved. These resonance parameters were in agreement with the distorted octahedral coordination of Cu^II^ ions in CuO clusters.^29^ This result suggested the existence of a high distribution of CuO clusters, which explained the invisible copper species in HRTEM. This result was also consistent with the Cu 2p XPS analysis. Upon 365 nm LED illumination, the intensity of the Cu^II^ signal was expected to decrease if the Cu^II^ ions could accept electrons to form EPR‐silent Cu^I^ sites.^29^ However, the spectra under chopped light almost overlapped, indicating the photoinduced electrons were trapped by Pt rather than the CuO sites (Figure 3 d). Thus, the introduction of Pt was important to impede charge recombination on CuO_*x*_ clusters, thus resulting in improved performance of Cu_0.1_Pt_0.5_/PC‐50.

**Figure 3:**
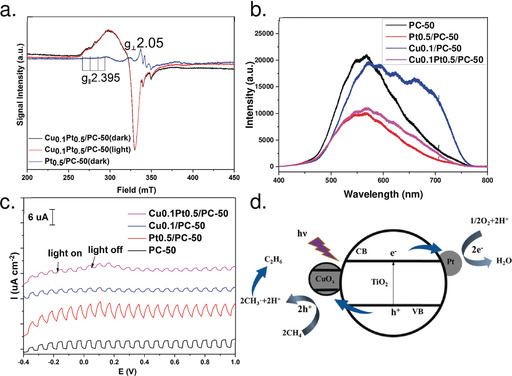
a) EPR spectra of Cu_0.1_Pt_0.5_/PC‐50 (light on and light off) and Pt_0.5_/PC‐50 (light off). b) PL spectra of Cu_0.1_Pt_0.5_/PC‐50, Pt_0.5_/PC‐50, Cu_0.1_/PC‐50, and PC‐50. c) Photocurrent of Cu_0.1_Pt_0.5_/PC‐50, Pt_0.5_/PC‐50, Cu_0.1_/PC‐50, and PC‐50. d) Proposed photocatalytic OCM process over Cu_0.1_Pt_0.5_/PC‐50.

The facilitation of charge transfer was further investigated by PL spectroscopy (Figure 3 b). An obvious band could be observed for the pristine PC‐50, while the PL intensity decreased notably after the incorporation of Pt nanoparticles. This result suggested the efficient separation of photoinduced electrons and holes by Pt nanoparticles. In the case of Cu_0.1_/PC‐50, a photoluminescence spectrum with fine structure was shown, which could be attributed to the highly dispersed copper species.^30^ According to the UV/Vis DRS result, it was suggested that the photoexcitation occurred by charge transfer from oxygen to copper in the clusters. Considering the enhanced absorption in the UV region observed in UV/Vis DRS spectra and the larger enhanced emission in the PL spectra, the photoinduced carriers in PC‐50 probably recombined in the CuO_*x*_ clusters over Cu_0.1_/PC‐50. This hypothesis was also consistent with the analysis of the band structure mentioned above and in Figure 3 d. More importantly, the PL intensity of Cu_0.1_Pt_0.5_/PC‐50 was significantly lower than that of Cu_0.1_/PC‐50, thus indicating that the photoinduced electrons in PC‐50 were transferred to Pt rather than to the CB of CuO_*x*_ clusters.

The function of Pt as an electron sink was further consolidated by the transient photocurrent response (Figure 3 c). As compared with pristine PC‐50, Pt_0.5_/PC‐50 exhibited higher reduction photocurrent density because of the efficient transfer of electrons to Pt nanoparticles, whereas the introduction of copper species resulted in a lower photocurrent response for both Cu_0.1_/PC‐50 and Cu_0.1_Pt_0.5_/PC‐50. As mentioned above, the valence bands of CuO and Cu_2_O were less positive than that of TiO_2_. This decay of photocurrent density could be explained by the weak oxidative potential of photoinduced holes on CuO_x_ clusters.^15^

Based on the above characterisations and investigations, a probable mechanism of photocatalytic OCM over Cu_0.1_Pt_0.5_/PC‐50 was proposed (Figure 3 d). Upon light irradiation, electrons could be excited from the VB of PC‐50 to its CB and then migrate to Pt, while holes could be transferred to the VB of CuO_*x*_ clusters. This process not only retarded the recombination of photoinduced electrons and holes, but also lowered the oxidation potential of photoinduced holes to avoid deep dehydrogenation and overoxidation. A C−H bond in CH_4_ molecules was cleaved by the holes in the VB of CuO_*x*_ clusters to form methyl radicals and protons. The combination of methyl radicals formed ethane molecules, and deep dehydrogenation could lead to the formation of ethylene. O_2_ could be reduced by electrons from Pt nanoparticles to form O_2_^.−^, and the protons could be removed by O_2_^.−^ to form water. The synergy effects between Pt and CuO_x_ clusters at reduction sites and oxidation sites, respectively, were highlighted to complete the catalytic cycle.

In summary, we have reported the first example of a continuous photocatalytic OCM process at room temperature and atmospheric pressure in a flow system. The Pt nanoparticles and CuO_x_ clusters were introduced onto PC‐50 by photodeposition and wet impregnation methods, respectively. The separation of photoinduced e^−^/h^+^ was facilitated and the oxidation potential of holes was lowered to avoid overoxidation, leading to high yield and selectivity towards C_2_ hydrocarbons. The synergy of Pt nanoparticles and CuO_*x*_ clusters resulted in the increased C_2_ yield (6.8 μmol h^−1^), which was approximately 3.5 times as high as that observed with PC‐50 and more than twice as high as the sum of the activity of Pt/PC‐50 (1.07 μmol h^−1^) and Cu/PC‐50 (1.9 μmol h^−1^), resulting in an AQE of 0.5 % at 365 nm. The selectivity of 60 % was also comparable to that of traditional OCM thermal catalysts, and the high photocatalytic activity remained stable after a long experimental period. Overall, this study provides an effective green route for methane upgrade.

## Conflict of interest

The authors declare no conflict of interest.

## Supporting information

As a service to our authors and readers, this journal provides supporting information supplied by the authors. Such materials are peer reviewed and may be re‐organized for online delivery, but are not copy‐edited or typeset. Technical support issues arising from supporting information (other than missing files) should be addressed to the authors.

Supplementary
