# Supplementary material for: Platinum‐ and CuOx‐Decorated TiO2 Photocatalyst for Oxidative Coupling of Methane to C2 Hydrocarbons in a Flow Reactor
Source: Angew Chem Int Ed Engl. 2020 Jul 16;59(44):19702–7. doi: 10.1002/anie.202007557 (PMC7689917; doi:10.1002/anie.202007557)
Supplement: Supplementary file 1 — Supplementary [file ANIE-59-19702-s001.pdf]

Angewandte Chemie
Eine Zeitschrift der Gesellschaft Deutscher Chemiker

Supporting Information

Platinum- and CuO_x-Decorated TiO_2 Photocatalyst for Oxidative Coupling of Methane to C_2 Hydrocarbons in a Flow Reactor

*Xiyi Li, Jijia Xie, Heng Rao, Chao Wang, and Junwang Tang**

anie_202007557_sm_miscellaneous_information.pdf

Supporting Information

©Wiley-VCH 2019
69451 Weinheim, Germany

Table of Contents

Experimental Procedures 3
The diagram of the reactor 5
Additional charaterizations 6
Control experiments 10
Comparison of the performance of photocatalytic methane conversion 11
Reference 12

Experimental Procedures

Synthesis of Cu_x/PC-50: Cu was introduced by a wet impregnation method. In a typical experiment, different concentration of $Cu(NO_3)_2·2.5H_2O$ (98%, Alfa Aesar) dissolved in 150 µL deionized (DI) water was added to 200 mg of TiO_2 (Millennium PC-50) under vigorous stirring in an alumina crucible. After drying at 70 ℃ for 3 h, the crucible was equipped with a lid and transferred to a muffle furnace (Carbolite, CWF 1300). The mixture was calcined at 400 ℃ for 4 h with a heating rate of 5 ℃/min. The samples synthesized by this impregnation method was denoted as Cu_x/PC-50 (x = 0.05, 0.1, 0.2, 0.3, 0.4 $_{wt.}$%, corresponding to the loading weight percentage of Cu at TiO_2).

Synthesis of $Pt_{0.5}$/PC-50: Pt was introduced by a photodeposition method. Typically, 250 mg TiO_2 was dispersed into a solution of 27 mL DI water and 3 mL methanol. Then, 0.5$_{wt.}$% (the weight percentage of Pt to TiO_2) of H_2PtCl_6 (99.995%, Sigma-Aldrich) was added to the above solution and allowed to be bubbled with argon (99.999%, BOC) for 20 minutes. Afterwards, the suspension was stirred under irradiation with a Multichannel 365 nm LED (Perfect Light, Beijing) at 25 ℃ for 3h. The samples were collected by centrifugation, washed with DI water for three times and dried at 70 ℃ for 6 h.

Synthesis of $Cu_{0.1}Pt_y$/PC-50: Pt was introduced by photodeposition method and then Cu was incorporated by wet impregnation method. Typically, 250 mg TiO_2 was dispersed into a solution of 27 mL DI water and 3 mL methanol. Then, a different amount (0.1~2.0 $_{wt.}$ %, the weight percentage of Pt to TiO_2) of H_2PtCl_6 was added to the above solution and allowed to be bubbled with argon for 20 minutes. Afterwards, the suspension was stirred under irradiation with a Multichannel 365 nm LED at 25 ℃ for 3h. The samples were collected by centrifugation, washed with DI water for three times and dried at 70 ℃ for 6 h. The samples obtained here were denoted as Pt_y/PC-50-unheating (y = 0.1, 0.5, 1.0, 1.5, 2.0). The following deposition of Cu was similar to the procedure mentioned in the preparation of Cu_x/PC-50, except the change of precursor from PC-50 to Pt_y/PC-50 (y = 0.1, 0.5, 1.0, 1.5, 2.0).

Synthesis of $Pt_{0.5}Cu_{0.1}$/PC-50: The synthesis of $Pt_{0.5}Cu_{0.1}$/PC-50 was similar to the synthesis of $Cu_{0.1}Pt_{0.5}$ /PC-50 just changing the order of two components preparation.

Heating treatment: In order to maintain the consistent experimental condition, $Pt_{0.5}$/PC-50-unheating and TiO_2 were also heated at muffle furnace with the program used in the preparation of Cu_x/PC-50. They were denoted as $Pt_{0.5}$/PC-50 and PC-50, respectively in the following section without special note.

Material Characterization: The powder X-ray diffraction (XRD) patterns were measured in a Stoe STADI-P instrument (2° to 40°, step 0.5 ° at 5.0 s/step) using Mo Kα1 (wavelength 0.70930 Å, 50 kV and 30 mA). Ultraviolet-visible diffuse reflectance spectroscopy (UV-Vis DRS) measurements were conducted by an Agilent Carry 3500 UV-Vis-NIR spectrophotometer with a diffuse reflectance unit. Raman spectroscopy was conducted at Renishaw InVia Raman with 514 nm excitation laser, ranging from 100 – 1200 cm^{-1}. X-ray photoelectron spectroscopy (XPS) measurements were performed using a Thermoscientific XPS K-alpha and the obtained results were analyzed by Casa XPS software. A JEOL JSM-7401F SEM equipped with Energy Dispersive-X-Ray (EDX) was applied to analyze the micro-morphology and composition

information of the materials on carbon tips. The transmission electron microscopy (TEM) images, high-resolution transmission electron microscopy (HRTEM) images were obtained using a JEOL 2010 instrument. Photoluminecscence (PL) spectra were collected by Renishaw InVia Raman with 325 nm excitation laser ranging from 200 – 800 nm. Electron paramagnetic resonance (EPR) was measured by a MS-5000 spectrometer at ambient temperature (288 K). Powder samples (~30 mg) were weighed in quartz tubes and sealed with septa. Microwave power was set at 100 microwatts, while the frequency was 9.485 GHz. A 365 nm LED (80 mW cm^{-2}) was applied for the experiment under irradiation.

Photo-electrochemical test: The electrode was prepared by dispersion of viscous pastes of pristine TiO_2 or modified TiO_2 onto fluorine doped tin oxide (FTO). Typically, 30 mg TiO_2 or modified TiO_2, 60 mg of ethyl cellulose, 6 drops of terpineol and 1 ml ethanol were mixed together and ground for 10 minutes to prepare pastes. The coating process was kept at 70 ℃ to improve their adhesion. The total exposed area of the film was 1.0 cm^2. The transient photocurrent response was investigated over a three-electrode electrolytic cell with an electrochemical workstation (IVIUM). The FTO electrode with catalysts was installed as a working electrode, the Pt plate as the counter electrode and Ag/AgCl electrode was applied as the reference electrode in 0.1 M Na_2SO_4 electrolyte. The light source was provided by a 150 W Xenon lamp with 1.5 AM filter and the switching period was set as 3 s.

Photocatalytic activity tests: The photocatalytic activity of the as-synthesized samples toward methane conversion was investigated in a flow reactor (Figure S1) connected with a GC. Typically, 100 mg photocatalyst was weighed and dispersed in 50 mL de-ionised water. The suspension was allowed to be sonicated for 15 minutes. Then, the suspension was filtered with a glass fibre membrane (diameter 37 mm, pore size 0.22 μm) to produce a uniform film, which was dried at 70 ℃ for 12 hours. After drying, the film was fixed in the reactor using a stainless ring and sealed with a rubber O-ring. A temperature probe was inserted from the bottom of the reactor in order to monitor the temperature of the reaction. The flow rate and ratio of different gases (air, methane, argon, BOC 99.999%) were adjusted by three mass flow controller (MFC, Bronkhorst). The film was then irradiated by 365 nm light by a 40 W LED (Beijing Perfect Light, PLS-LED 100). For each experiment, the system was allowed to purge with gases at an expected ratio to reach the equilibrium of adsorption-desorption before testing. The outlet gases were detected by an Agilent 7820 GC equipped with an online injection valve at regular time intervals (30 minutes). In this GC, two columns (CarbonPlot and Molecular 5A) are connected with thermal conductivity detectors (TCD) to analyze H_2, O_2, N_2, CH_4, respectively. Another column (HP-PLOT Q) is linked with flame ionization detectors (FID) to analyze organic products, such as C_2H_4, C_2H_6, CH_3OH, C_2H_5OH, respectively. The outlet was also connected to another GC (Varian 450) by a long tube. A FID equipped with methanizer in this GC was used to quantify the concentration of CO_2 and CO.

Calculation of selectivity: The main products were C_2H_6, C_2H_4, CO_2 and a tiny amount of other products. Thus, the selectivity for C_2 products were calculated as below:

Selectivity% = 100% × 2 × total moles of C_2H_6 and C_2H_4 / (2 × total moles of C_2H_6 and C_2H_4 + obtained moles of CO_2)

Calculation of apparent quantum efficiency (AQE) based on the conversion of methane:

$$AQE = \frac{2\times number\ of\ C2H6\ molecules+4\times number\ of\ C2H4\ molecules+8\times number\ of\ CO2\ molecules}{Number\ of\ incident\ phototons} \times \mathbf{100}\%$$

The irradiation area is 10 cm^2; The light intensity of 40 W 365 nm LED is 160 mW cm^{-2}.

The diagram of the reactor

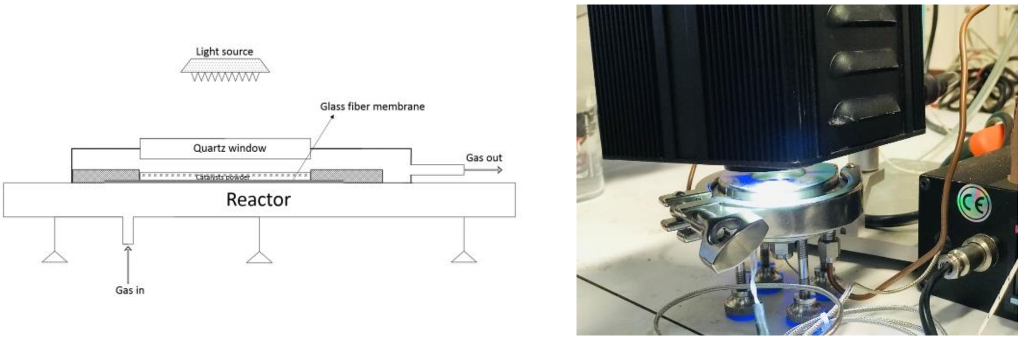

Figure S1. The flow reactor for photocatalytic OCM reaction

The house-design gastight flow reactor used in this study was made from stainless-steel equipped with a quartz window on its top made by Beijing Perfect Light Ltd. The catalyst film was fixed by a stainless-steel ring at the middle high of the reactor. A thermo senser was inserted at the bottom the reactor bed to monitor the reaction temperature. Mixed reactant gas flowed into the reactor at the bottom of t[illegible]eactor, then through the catalyst bed and finally flowed into a GC for products analysis.

Additional Characterizations

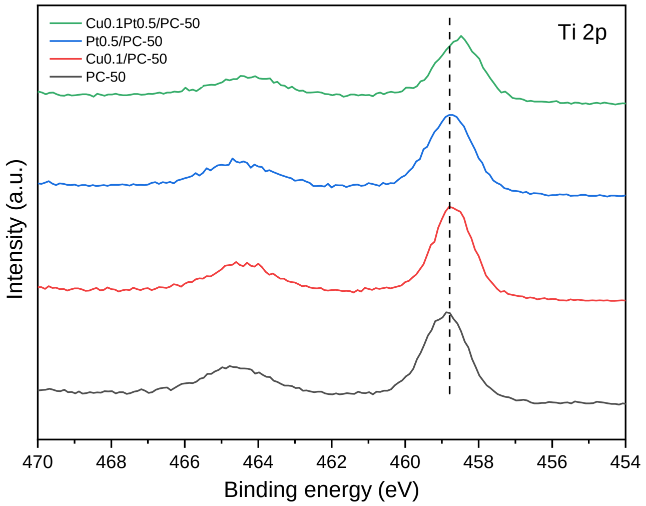

Figure S2. Ti 2p XPS spectra of $Cu_{0.1}Pt_{0.5}$/PC-50, $Pt_{0.5}$/PC-50, $Cu_{0.1}$/PC-50 and PC-50

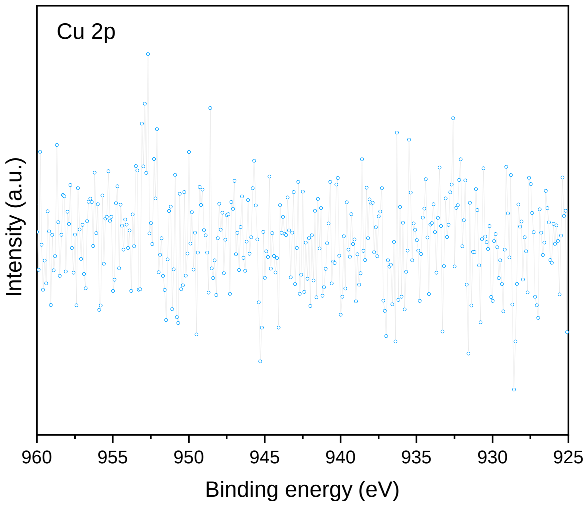

Figure S3. Cu 2p XPS spectra of $Cu_{0.1}Pt_{0.5}$/PC-50

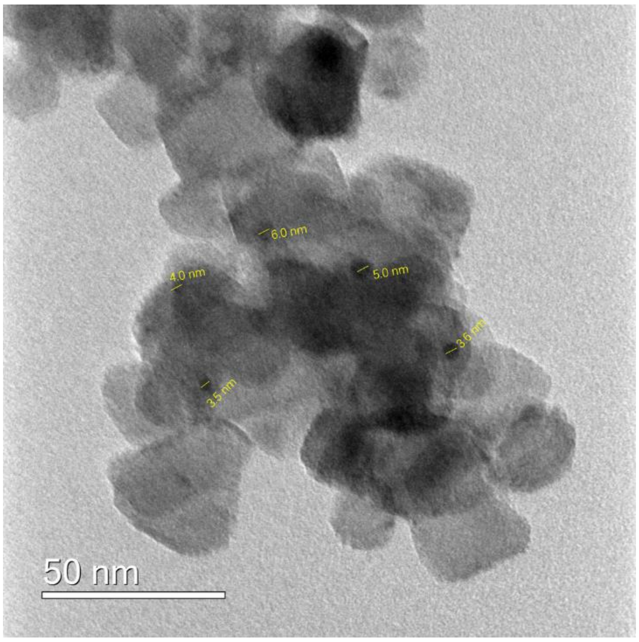

Figure S4. TEM images of $Cu_{0.1}Pt_{0.5}$/PC-50

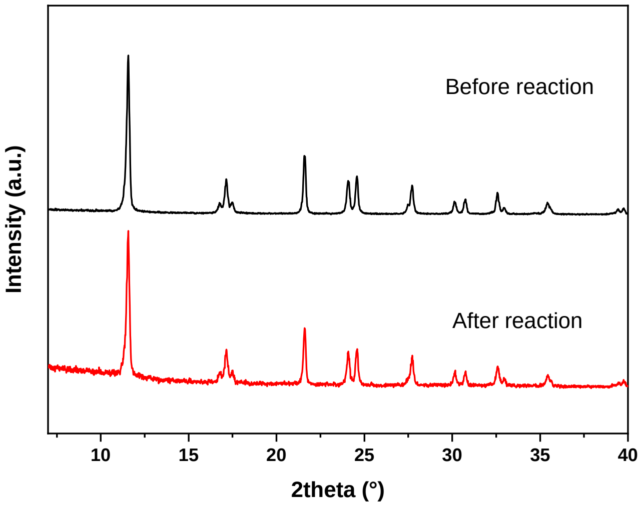

Figure S5. PXRD spectra of $Cu_{0.1}Pt_{0.5}$/PC-50 before and after reaction

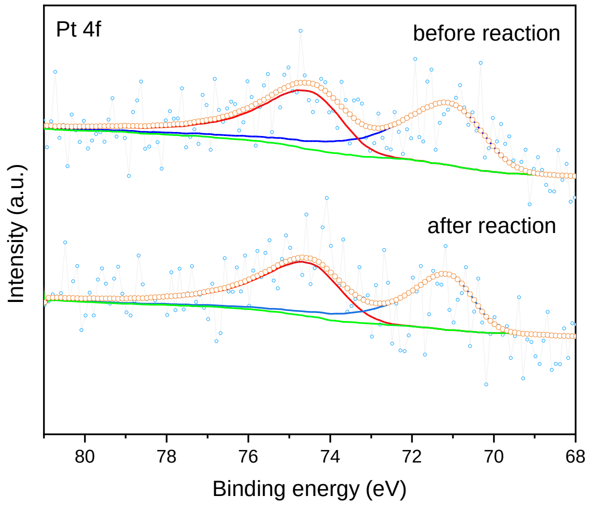

Figure S6. Pt 4f XPS spectra of $Cu_{0.1}Pt_{0.5}$/PC-50 before and after the reaction

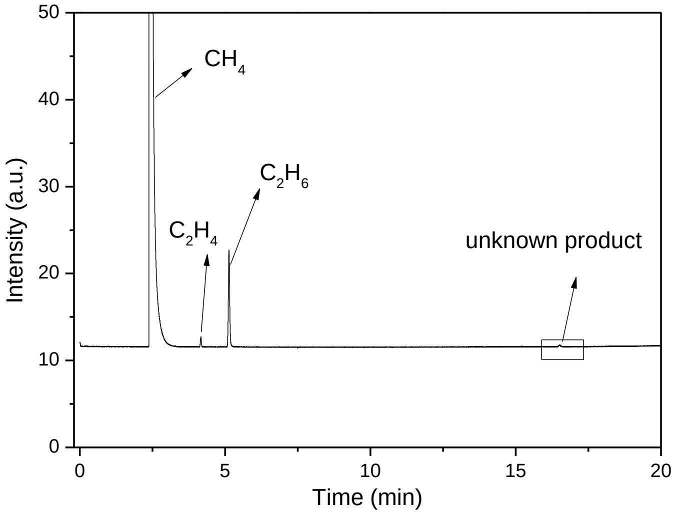

Figure S7. The zoom-in of FID spectra during photocatalytic reaction of $Cu_{0.1}Pt_{0.5}$/PC-50

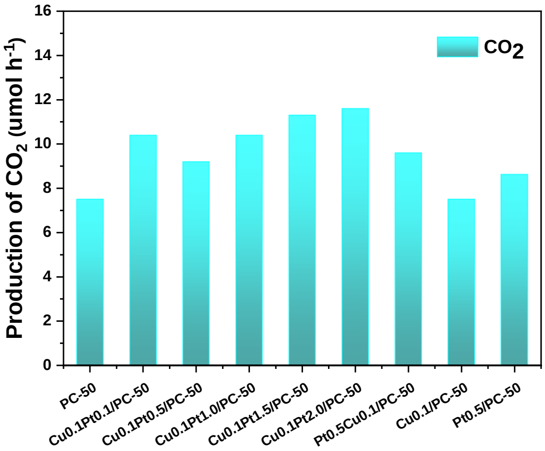

Figure S8. CO_2 prodution over $Cu_{0.1}Pt_y$/PC-50 (y = 0.1, 0.5, 1.0, 1.5, 2.0 $_{wt}$%), $Cu_{0.1}$/PC-50, PC-50, $Pt_{0.5}$/PC-50 and $Pt_{0.5}Cu_{0.1}$/PC-50; (Reaction condition: O_2 : CH_4 = 1 : 400, GHSV = 2400 h^{-1}, 10% of CH_4, 365 nm LED 40 W, 40 ℃)

Investigation of optimal amount of catalysts

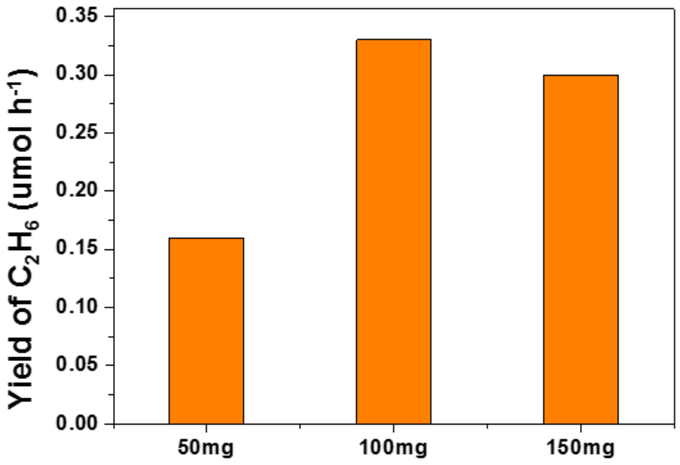

Figure S9 Photocatalytic OCM process over TiO_2 with different amount of catalysts

(Reaction condition: O_2: CH_4 = 1: 4000, 1% CH_4, total flow rate 50 mL min^{-1}, 365 nm LED 20 W, 30℃)

TiO_2 without modification was selected as a model catalyst to investigate optimal amount of catalysts for our photocatalytic OCM system, as shown in this Figure The yield of C_2H_6 remained unchanged after the amount increasing from 50 mg to 100 mg (linear correlation), while it decreased after further increasing to 150 mg. This decrease might be due to light scattering by extra TiO_2.

Control experiments

Table S1. Control experimental results of the photocatalytic OCM over $Cu_{0.1}Pt_{0.5}$/PC-50

Order	CH_4	LED	Products
1	×	√	×
2	√	×	×

Comparison of the performance of photocatalytic methane conversion

Table S2. Representative works on photocatalytic methane conversion to ethane/ethane at room temperature and atmospheric pressure

Samples	Reaction type	Rector type	Conditions	Yield rate of C_2H_4 and C_2H_6	References
$Cu_{0.1}Pt_{0.5}$/PC-50	OCM	Flow reactor	Room temperature; 40 W 365 nm LED; 0.1 g catalyst; O_2 : CH_4 = 1 : 400, GHSV = 24000 g^{-1} h^{-1}, 10% of CH_4;	6.8 µmol h^{-1}	This work
ZrO_2/SiO_2(0.1)	NOCM	Batch reactor	310 K; Reaction time 3 h; 0.5 g catalyst; 200 µmol CH_4	0.0365 µmol h^{-1}	[1]
MgO	Methane oxidation	Batch reactor	293 K; High pressure mercury lamp; Reaction time 3 h; 399.9 Pa N_2O; 4.2 µmol CH_4	0.00268 µmol h^{-1}	[2]
MgO/SiO_2(2.0)	NOCM	Batch reactor	310 K; 300 W Xe lamp; Reaction time 3 h; 0.2 g catalyst; 200 µmol CH_4	0.0092 µmol h^{-1}	[3]
Au/ZnO nanosheets	NOCM	Batch reactor	Ambient temperature; 300 W Xe lamp; Reaction time 4 h; 0.001 g catalyst; 22.3 µmol CH_4	0.0113 µmol h^{-1}	[4]
0.5%Pt/TiO_2	Methane oxidation	Batch reactor	298 K; Phiplips UV lams (TUV 4W/G4 T5); Reaction time 6 h; 0.075 g catalyst; 80 mL CH4	2.08 µmol h^{-1}	[5]
Ga_2O_3-K	NOCM	Batch reactor	Room temperature; 300 W Xe lamp; Reaction time 3 h; 0.2 g catalyst; 200 µmol CH_4	0.054 µmol h^{-1}	[6]
SiO_2-Al_2O_3-TiO_2	NOCM	Batch reactor	Room temperature; 250 W Xe lamp; Reaction time 3 h; 1.0 g catalyst; 200 µmol CH_4	0.74 µmol h^{-1}	[7]
(Zn^+, Zn^{2+})-ZSM-5	NOCM	Batch reactor	303 K; 150 W high-pressure Hg lamp; Reaction time 8 h; 1.0 g catalyst; 200 µmol CH_4	2.988 µmol h^{-1}	[8]
Pt/Ga-TiO_2-SiO_2	NOCM	Batch reactor	333 K; 300 W Xe lamp; Reaction time 4 h; 0.2 g catalyst; 44.6 µmol CH_4	0.314 µmol h^{-1}	[9]
Ce(0.5)/Al_2O_3	NOCM	Batch reactor	310 K; 300 W Xe lamp; Reaction time 3 h; 0.2 g catalyst; 200 µmol CH_4	0.1178 µmol h^{-1}	[10]
FSM-16	NOCM	Batch reactor	310 K; 300 W Xe lamp; Reaction time 3 h; 0.2 g catalyst; 200 µmol CH_4	0.018 µmol h^{-1}	[11]
Ga-ETS-10-0.2	NOCM	Batch reactor	303 K; 150 W high-pressure Hg lamp; Reaction time 5 h; 0.2 g catalyst; 200 µmol CH_4	2.3 µmol h^{-1}	[12]
1.5%Pd/TiO_2	Methane oxidation	Batch reactor	298 K; Phiplips UV lams (254 nm, TUV 4W/G4 T5); Reaction time 6 h; 0.075 g catalyst; 80 mL CH_4	2.13 µmol h^{-1}	[13]
Ag-HPW/TiO_2	Photochemical coupling of methane	Batch reactor	Ambient temperature; 400 W Xe lamp; Reaction time 7 h; 0.1 g catalyst; 0.3 MPa CH_4	2.3 µmol h^{-1}	[14]

References

[1] H. Yoshida, M. G. Chaskar, Y. Kato, T. Hattori, *Chem. Commun.* **2002**, *2*, 2014–2015.

[2] C. Yun, M. Anpo, Y. Mizokoshi, Y. Kubokawa, *Chem. Lett.* **1980**, *9*, 799–802.

[3] L. Yuliati, T. Hattori, H. Yoshida, *Phys. Chem. Chem. Phys.* **2005**, *7*, 195.

[4] L. Meng, Z. Chen, Z. Ma, S. He, Y. Hou, H.-H. H. Li, R. Yuan, X.-H. H. Huang, et al., *Energy Environ. Sci.* **2018**, *11*, 294–298.

[5] L. Yu, Y. Shao, D. Li, *Appl. Catal. B Environ.* **2017**, *204*, 216–223.

[6] L. Yuliati, T. Hattori, H. Itoh, H. Yoshida, *J. Catal.* **2008**, *257*, 396–402.

[7] H. Yoshida, N. Matsushita, Y. Kato, T. Hattori, *J. Phys. Chem. B* **2003**, *107*, 8355–8362.

[8] L. Li, G.-D. Li, C. Yan, X.-Y. Mu, X.-L. Pan, X.-X. Zou, K.-X. Wang, J.-S. Chen, *Angew. Chemie Int. Ed.* **2011**, *50*, 8299–8303.

[9] S. Wu, X. Tan, J. Lei, H. Chen, L. Wang, J. Zhang, *J. Am. Chem. Soc.* **2019**, *141*, 6592–6600.

[10] L. Yuliati, T. Hamajima, T. Hattori, H. Yoshida, *J. Phys. Chem. C* **2008**, *112*, 7223–7232.

[11] L. Yuliati, M. Tsubota, A. Satsuma, H. Itoh, H. Yoshida, *J. Catal.* **2006**, *238*, 214–220.

[12] L. Li, Y.-Y. Cai, G.-D. Li, X.-Y. Mu, K.-X. Wang, J.-S. Chen, *Angew. Chemie Int. Ed.* **2012**, *51*, 4702–4706.

[13] L. Yu, D. Li, *Catal. Sci. Technol.* **2017**, *7*, 635–640.

[14] X. Yu, V. L. Zholobenko, S. Moldovan, D. Hu, D. Wu, V. V Ordomsky, A. Y. Khodakov, *Nat. Energy* **2020**, DOI 10.1038/s41560-020-0616-7.
